# Insight View on the Role of in Ovo Feeding of Clenbuterol on Hatched Chicks: Hatchability, Growth Efficiency, Serum Metabolic Profile, Muscle, and Lipid-Related Markers

**DOI:** 10.3390/ani11082429

**Published:** 2021-08-18

**Authors:** Ahmed A. Saleh, Rashed A. Alhotan, Abdulrahman S. Alharthi, Eldsokey Nassef, Mohamed A. Kassab, Foad A. Farrag, Basma M. Hendam, Mohamed M. A. Abumnadour, Mustafa Shukry

**Affiliations:** 1 Department of Poultry Production, Faculty of Agriculture, Kafrelsheikh University, Kafrelsheikh 33516, Egypt; 2 Department of Animal Production, College of Food and Agriculture Sciences, King Saud University, P.O. Box 2460, Riyadh 11451, Saudi Arabia; ralhotan@ksu.edu.sa (R.A.A.); Abalharthi@ksu.edu.sa (A.S.A.); 3 Department of Nutrition and Clinical Nutrition, Faculty of Veterinary Medicine, Kafrelsheikh University, Kafrelsheikh 33516, Egypt; dsokeynassef@yahoo.com; 4 Department of Histology, Faculty of Veterinary Medicine, Kafrelsheikh University, Kafrelsheikh 33516, Egypt; kassabkassab2000@yahoo.com; 5 Department of Anatomy and Embryology, Faculty of Veterinary Medicine, Kafrelsheikh University, Kafrelsheikh 33516, Egypt; foad.farrag@vet.kfs.edu.eg; 6 Genetics and Genetic Engineering, Department of Husbandry and Development of Animal Wealth, Faculty of Veterinary Medicine, Mansoura University, Mansoura 35516, Egypt; basmahedam@mans.edu.eg; 7 Department of Anatomy and Embryology, Faculty of Veterinary Medicine, Alexandria University, Edfina 22756, Egypt; m.abumandour@yahoo.com; 8 Department of Physiology, Faculty of Veterinary Medicine, Kafrelsheikh University, Kafrelsheikh 33516, Egypt

**Keywords:** clenbuterol, in ovo feeding, growth performance, myogenic genes, lipid indices, embryo chicks

## Abstract

**Simple Summary:**

This study examined the effects of ovo injection of clenbuterol on fat deposition and growth performance in chickens, which is prejudicial to poultry consumers and muscle growth-related genes, egg hatchability, and fertility. The achieved result showed a definite effect of clenbuterol on body gain and hatchability. It decreased fat deposition and upregulation of muscle growth-related gene expressions accompanied by modulation of fatty and amino acid composition, reflecting a new insight into the intracellular pathways of clenbuterol supplementation on chicks.

**Abstract:**

The present study aimed to assess the in ovo administration of clenbuterol on chick fertility, growth performance, muscle growth, myogenic gene expression, fatty acid, amino acid profile, intestinal morphology, and hepatic lipid-related gene expressions. In this study, 750 healthy fertile eggs from the local chicken breed Dokki-4 strain were analyzed. Fertile eggs were randomly divided into five experimental groups (150 eggs/3 replicates for each group). On day 14 of incubation, in addition to the control group, four other groups were established where 0.5 mL of worm saline (30 °C) was injected into the second group of eggs. In the third, fourth, and fifth groups, 0.5 mL of worm saline (30 °C), 0.9% of NaCl, and 10, 15, and 20 ppm of clenbuterol were injected into the eggs. Results suggested that clenbuterol increased growth efficiency up to 12 weeks of age, especially at 15 ppm, followed by 10 ppm, decreased abdominal body fat mass, and improved hatchability (*p* < 0.01). Clenbuterol also modulated saturated fatty acid levels in the breast muscles and improved essential amino acids when administered at 10 and 15 ppm. Additionally, clenbuterol at 15 ppm significantly decreased myostatin gene expression (*p* < 0.01) and considerably increased *IGF1r* and IGF-binding protein (*IGFBP*) expression. Clenbuterol administration led to a significant upregulation of hepatic PPARα, growth hormone receptor, and Lipoprotein lipase (*LPL*) mRNA expression with a marked decrease in fatty acid synthase (*FAS*) and sterol regulatory element-binding protein 1 (*SREBP-1c*) expression. In conclusion, the current study revealed that in ovo injection of clenbuterol showed positive effects on the growth of hatched chicks through reduced abdominal fat deposition, improved intestinal morphology, and modulation of hepatic gene expressions in myogenesis, lipogenesis, and lipolysis.

## 1. Introduction

A significant proportion of high-quality meat supply is poultry. Recently, poultry meat consumption has increased; therefore, poultry meat prices are considerably more competitive than those of other species, especially chicken broilers [1]. In ovo fertilized egg feeding with bioactive substances has recently been extensively used in poultry growth and production concerning improvements in nutrigenomics, disease resistance, and biology [2].

In ovo feeding of such substances influenced the pre- and post-hatching physiological status of broiler embryos, leading to improved hatchability, the superior nutritional quality of hatchlings, greater vigor, and higher post-hatch growth [3]. This procedure also improved chick immunity and increased disease resistance, resulting in low mortality and increased hatchability and fertility in post-hatching [4], helping to increase the growth gain [5,6]. Additives of feed, including β2-adrenergic agonists, have been used to enrich carcass quality and lower feed requirements for animals with increased muscle mass concerning fat [7]. Clenbuterol is a β2-adrenergic agonist that induces muscle hypertrophy [8]. Cyclic adenosine monophosphate can increase the fat ratio [9], resulting in increased muscle size coupled with an increased protein level [10,11].

Clenbuterol is widely used in farm animals for respiratory disorders and enhances protein creation [12]. These compounds are also used as growth promoters [13]. However, because of the possibility that the residues of these drugs in food would be detrimental to human health, the European Union has banned their use in animal production [14]. Clenbuterol can be absorbed by the liver, lungs, kidneys, and pancreas when orally administered in animals [15]. Clenbuterol is cleared gradually, with liver, eye, hair, and feathers displaying the slowest degradation of residues [16]. Abated use of clenbuterol has been observed in the Netherlands, Germany, and Northern Ireland. Clenbuterol is the only beta-agonist registered for animals in most European countries [17]. In the Netherlands, the approved oral or parenteral doses of clenbuterol are 1.5 mg/kg body weight for ten days for the treatment of bronchospasm in horses and cattle and a single dose of 300 mg/kg body weight (±0.5 mg/kg body weight) for tocolysis [12]. Some European authorities have introduced a national maximum residue level (MRL) of 0.5 mg/kg of clenbuterol/kg in edible tissue, such as the United Kingdom and The Netherlands, with a 1 mg/kg liver [12]. Recent research suggests a beneficial impact of lower doses of clenbuterol on the carcass’s growth and quality [12]. Some studies have used clenbuterol [18]; they showed that clenbuterol burns more body fat. Besides, Emili et al. [19] proved that Clenbuterol neonatal treatment enhances spinogenesis. Mohamed et al. [20] reported that clenbuterol at two doses, five ppm and ten ppm/kg diet for one month, boosts growth and reduces fat deposition rates on Nile tilapia. Clenbuterol feeding lessens the abdominal body fat in hatched chicks [21,22]. On the same lines, Buyse et al. [23] demonstrated that clenbuterol at (42 ppm) from day 1 to 2 or from 4 weeks of age until slaughter age, acts primarily on fat deposition at a single intraperitoneal (i.p.) feeding of clenbuterol (0.1 mg/kg body weight) in the broiler. In addition, Ocampo et al. [24] reported that low-use doses of clenbuterol are given to chickens to alleviate ascites syndrome. Besides, Zhou and Han [25] showed the impacts of dietary clenbuterol on the quality of duck carcasses at doses of CL at 0.5, 1, 2, 3, and 5 mg/kg. On the other hand, Dupont-Versteegden et al. [26] reported that, in addition to its positive effects on muscle growth, long-term clenbuterol treatment may have negative side effects such as increased muscle fatigue and deformities. In addition [27] reported that the abuse of clenbuterol by individuals lead to immunosuppression.

Although poultry signifies a critical source of protein worldwide, with comparatively minimal costs and a high processing ratio for feed [28], it contains 13–14.5% fat [29], most of which is not required. The fat buildup causes production problems [30], leading to fatty liver and increased mortality [31]. Therefore, in this study, we assessed the effects of clenbuterol on growth parameters and antioxidant, physiological, and immunity markers, performed transcriptomic analysis of lipid-related and myogenic gene expression levels in the liver and muscle, and assessed the effect of in ovo clenbuterol feeding on the intestinal morphometry and villi length, bursa of Fabricius, and spleen.

## 2. Materials and Methods

According to the Egyptian code of ethics, this experiment was performed and approved by the animal ethics committee at the Kafrelsheikh University, Egypt.

### 2.1. Experimental Design

Clenbuterol was provided by Sigma (St. Louis, MO, USA). A total of 900 incubating eggs from local chicken breeds, the Dokki-4 strain were obtained from the Research Station, Sakha, Institute for Animal Development, Agricultural Research Centre, and Agriculture Ministry of Egypt. All eggs were placed large end up in an automatic incubator. In this study, 750 healthy fertile eggs that contained embryos were weighed and selected at seven days of incubation. Eggs were instantaneously cleaned, disinfected, and dried with soft tissue paper. The eggs were incubated at 36 °C–38 °C with a relative humidity of 55%. All eggs were placed large end up in an automatic turner. On day 14 of incubation, fertile eggs were randomly divided into five experimental groups of three replicates (50 eggs/replicate). An auto-needle hole using a 23-G needle with a depth of 25 mm was created at the broad end of each egg and 0.5 mL was injected in ovo-food solutions. In addition, a control group was used where 0.5 mL of worm saline (30 °C) was injected into the second group’s eggs. In the third, fourth, and fifth groups, eggs were injected with 0.5 mL of worm saline (30 °C), 0.9% NaCl, and clenbuterol at 10, 15, and 20 ppm/egg, respectively. After feeding, holes were filled with a nontoxic glue stick. At the end of 18 days of incubation, eggs were sprayed with TH4 disinfectant solution (2 mL/1000 mL of water) [32] and transferred to hatching trays at 37 °C with <70% moisture for the next three days. The time of hatching was confirmed. The incubation equipment consisted of an incubator (for the first 18 days) and hatcher (for the remaining three days until hatching). The eggs from each experimental group were placed in discrete and labeled containers in both the setter and hatcher. The live hatched chicks were counted after 21 days of incubation. Hatchability was assessed as a percentage of fertile eggs according to [33,34] using the following equations

Fertility (%) = (fertile eggs/total eggs) × 100.

Hatchability of the fertile eggs (%) = (hatched chick/fertile eggs) × 100.

A total of 200 healthy hatched chicks (40 birds/group with four replicates for each: control group, normal saline, clenbuterol 10, 15, and 20 ppm/egg) were selected and used for the feeding trial. The chicks grew for 12 weeks, and standard feed was provided for local chicken strains. Chicks were maintained in brooder pens for two weeks after hatching and transferred into separate locations. The chicks (8 birds/m^2^) were housed at the Kafrelsheikh University, Egypt, in an environmentally managed space. The house was kept at a temperature that was dependent on the bird’s age. The temperature was regulated through an air conditioner. During the experiment, air humidity was maintained at almost 70% [35]. The basal diet formulation was confirmed with the following [36] (Table 1). Body weight was measured at the nearest 0.1 g, and the ratio of feed conversion (FCR) was calculated. The birds were vaccinated against the common diseases in Egypt. Hitchner vaccine B1 (HB1) and Gumboro vaccine were administered through eye drops at the age of 7 and 10 days, respectively, and, at 13 days, intramuscular feedings of killed N.D.V., Reo, Gumboro, and infectious bronchitis vaccines were administered. Vaccines were intramuscularly injected with killed avian influenza viruses (AIV; H5N2) when they were 15 days of age, while Gumboro and LaSota were administered through eye drops at 20, 30 and 40 days of age. Subsequently, LaSota booster doses were administered at 51 days of age and then through an eye drop on a biweekly basis [37].

### 2.2. Growth Efficiency and Yield of Carcass

The experiment was finally completed at 12 weeks of age, 20 birds/group, five birds from each replicate per group were slayed for carcass yield estimation. The birds were weighed individually, and the FCR was calculated as the real consumption of feed (FI) divided by the body production. The slaughtering technique was performed following the Malaysian institute’s method [38]. The carcasses were sprayed, dipped cool at 2 °C for 30 min, and allowed to drain effectively for 5 min, and the carcass yields were measured as live body weight percentages. Abdominal fat was eliminated and evaluated according to Baziz et al. [39].

### 2.3. Blood and Tissue Sampling

At 12 weeks of age, two blood samples were obtained by wing vein puncture from five birds from each replicate, randomly selected from each group, under gentle restraint. One sample was obtained for hematological analysis. Non-heparinized syringes were used in one sample for serum collection, separated at 3000× *g*/15 min by centrifuge of the clotted blood at 4 °C and stored at −20 °C for additional biochemical analysis.

### 2.4. Hematological Analysis

Blood samples were used to assess the hemoglobin content (g/dL) with Drabkin’s technique utilizing the colorimetric form of cyanmethemoglobin after centrifugation [40]. Blood was smeared on a glass slide, left to dry, and then coated with Giemsa stain. Differential leukocyte counting was performed. One hundred leukocytes, including heterophils and lymphocytes, were counted on each blood film. H/L ratio was obtained by dividing the number of heterophils by the number of lymphocytes. Three slides were scored, and the means in each bird were calculated [41].

### 2.5. Blood Biochemical Analysis

The spectrophotometric analysis was conducted to evaluate global protein (g/dL), globulin, and albumin (g/dL) concentrations [42]. Biodiagnostic Company, Giza, Egypt, provided the commercial test kits. The lipoprotein fractions (VLDL, LDL, and HDL) were isolated using two sequential ultracentrifugation steps. the density was adjusted appropriately by adding NaCl (Sigma # S9888) and NaBr (Sigma # 310506), as detailed elsewhere [43] following [44]. In brief, plasma (5 mL) was transferred to quick seal tubes (Beckman Instruments, Palo Alto, CA, USA) and centrifuged for 18 h at 40,000 rpm, 4 °C in a 40.3 Ti fixed-angle rotor ultracentrifuge (Beckman, Brea, CA, USA). The 1.006 g/mL top fraction (VLDL) was brought back to a volume of 2.5 mL with saline (0.85%). The bottom fraction was adjusted to a relative of 1.063 with KBr and centrifuged for 18 h at 40000× g to obtain the LDL (top) and the HDL (bottom). After centrifugation, each lipoprotein sample was dialyzed extensively against Tris-buffered saline (TBS; 10 mM Tris-HCl, 140 mM NaCl, and 5 mM EDTA (pH 8.0)) for 24 h to remove NaBr. For each of the lipoproteins that were purified individually, Total lipid (mg/dL), triglyceride, cholesterol (mg/dL), high-density lipoprotein, lower-density lipoprotein (mg/dL) measurements were obtained using commercially available kits. As per the manufacturer’s instructions, maximum antioxidant efficiency was also calculated using commercial kits (Diamond Diagnostics). Serum aspartate aminotransferase (AST) and alanine aminotransferase (ALT) activities were determined [45].

### 2.6. Antioxidant Activity in Breast Muscles

Breast muscle samples in 100 mM cold potassium phosphate buffer, pH 7.2, were homogenized. Homogenate muscles were spun at 1500× *g* at 4 °C for 20 min, and the supernatant was added for further evaluation. The manufacturer’s protocol was used to calculate malondialdehyde concentration with a biodiagnostic kit (Biodiagnostic # MD 2529, Egypt). The biodiagnostic package (Biodiagnostic, # GP 2524, Egypt) was tested following the protocol for GSH-Px. The biodiagnostic kit (Biodiagnostic # SD 2521, Egypt) protocol was used to test SOD. MDA, GSH-Px, and SOD contents were measured at 534 nm, 340 nm, and 560 nm, respectively, using a UV–VIS spectrophotometer (NanoDrop One^C^, Thermo Scientific, Wilmington, DE, USA) using the software Excel 2016 (Microsoft, Redmond, WA, USA). 

### 2.7. Immunity Markers

Polymorphonuclear cell phagocytosis using Candida albicans was achieved according to [46]. The following aliquots were combined in the plastic tube: 100 μL fetal calf serum, 100 μL heat-killed C. Albicans (5 to 106/mL), and 100 μL blood. The tubes had been combined and incubated for 30 min at 37 °C, during which they were centrifuged for 5 min. The supernatant was removed, leaving a droplet in which the sediment was resuspended. Smears from the deposit were prepared, dried in the air, fixed with methyl alcohol, and stained with Giemsa stain. There were 200 heterophils examined, and the percentage of Candida-ingested heterophils was counted and expressed. A check for agarose gel cell lysis assessed serum lysosomal activity, as previously defined [47].

### 2.8. Amino Acid and Muscle Fatty Acid Profiles

Five birds from each replicate per group (20 birds/group) were selected to estimate muscle fatty acids and amino acid patterns. Extraction of fat was performed in the breast muscle using the chloroform–methanol (2:1) mixture to extract lipids, centrifuged for 10 min at 3000 rpm. The esterification procedure was achieved by adding the supernatant to 2 mL of methanol–sulfuric acid mix (95:5). The free fatty acids were provided by Sigma-Aldrich (Sigma, St. Louis, MO, USA). Fatty acid quantity was assessed using Agilent gas chromatography techniques (7890A GC). The flow rate conditions through the GC column and the splitless feeding mode were applied [48]. The amount of free amino acids (AA) in the breast muscle was evaluated [49]. Briefly, 2 g of muscle sample was homogenized with 20 mL of trichloroacetic acid (2%) for 2 min at 17.100 g. Subsequently, the homogenate was centrifuged at 3000× *g*/15 min, filtered through a 0.5 μm membrane, and dried. The derivatized samples and AA standards were inserted into the column for separation by high-performance liquid chromatography using a Nova-PakTM C18 column (4 μm, 3.9 × 4.6 mm).

### 2.9. Gene Expression Analysis

Total RNA was extracted from the tissue samples using the manufacturer’s easy-RED Total RNA Extraction Kits (iNtRON Biotechnology, Inc., Seongnam-Si, Korea). The RNA integrity was tested by agarose gel electrophoresis, and NanoDrop’s spectrophotometer was used to analyze the sample quantities. The first-strand cDNA was achieved by the HiSenScript cDNA package (iNtRON Biotechnology, Inc., Korea). The selected genes, with GAPDH as a standard gene, were amplified with specific primers and stable in the sample groups (Table 2). The mRNA expression was performed using the Stratagene MX3005P real-time PCR (Agilent Technologies, CA, USA) and the TOPreal™ PreMIX SYBR Green qPCR master blend (Enzynomics, Daejeon, Korea) as indicated by the manufacturer. Tools were used for MxPro QPCR. A 2−a technique, described above, was used to test the relative concentrations of gene expression. The relative intensities of gene expression were assessed using the 2−ΔΔct method as outlined in [50].

### 2.10. Histomorphometric Examination

Tissue samples from both ascending and descending limbs of the duodenum and the bursa of Fabricius and spleen were collected from five chickens from each treated group. The samples were fixed in 10% formaldehyde solution and then dehydrated in graded ethanol. The dehydrated samples were cleared in xylene and then embedded in paraffin. Moreover, 5-µm-thick paraffin-tissue sections were stained with hematoxylin and eosin. The stained sections were examined under a light microscope (Leica). The obtained images were subjected to morphometric analysis, including intestinal villi length of duodenum, total cell count of splenic parenchyma, and lymphoid follicles of the bursa of Fabricius using image analysis software (NIH, Bethesda, MD, USA). A total of 5 images from each bird were selected and the average was calculated (Mean ± SE) [57].

### 2.11. Data Analysis

Data analysis was performed using SPSS version 23 (IBM Corp, Armonk, NY, USA) [58]. A one-way study of variance accompanied by the multiple ranges of Duncan determined the significant difference between treatments at a *p*-value  <  0.05. Before conducting this test, Shapiro–Wilk and Levene’s experiments assessed normality. Polynomial contrasts were applied to find linear and quadratic impacts of different clenbuterol levels on the different parameters [59].

## 3. Results

### 3.1. Growth Performance Analysis and Carcass Traits

The effects of in ovo feeding on growth and carcass traits are shown in Table 3, in which the clenbuterol-injected group at 15 ppm showed higher (*p* = 0.023) weight gain and final body weight (*p* = 0.01) compared to other treated groups. Moreover, clenbuterol at 15 ppm improved the hatchability (*p* < 0.01) concerning normal saline and control groups and recovered the hatchability and fertility percentage of injected eggs. Additionally, there was a significant improvement in carcass yield (Table 3). Moreover, clenbuterol at 10 ppm also showed a markedly substantial difference in control and normal saline groups (*p* = 0.041). Furthermore, there was a significantly decreased abdominal fat weight percentage at 10 and 15 ppm dose of clenbuterol concerning other treated groups.

### 3.2. Blood Biochemical and Hematological Markers

There were no significant differences (*p* > 0.05) in RBC count, Hb level, and H/L ratio in different treated groups (Table 4). In ovo feeding of clenbuterol decreased the cholesterol, TG, total lipid, and LDL levels (*p* = 0.013, 0.016, 0.012, and 0.020 respectively) compared to other treated groups, with a noteworthy increase in HDL level (*p* = 0.011). Clenbuterol feeding resulted in considerably increased total protein and albumin levels compared to other treated groups. Moreover, clenbuterol had no considerable effect on AST and ALT levels. Additionally, clenbuterol groups showed a nonsignificant alteration in the total antioxidant capacity in other treated groups.

### 3.3. Immunity and Antioxidant Activity

MDA, GSH-PX, and SOD are presented in Table 5. The embryos’ results in ovo feeding with clenbuterol revealed normal antioxidant activities (GSH-PX and SOD; Table 4). There was no statistically significant difference in MDA level between different treated groups (*p* > 0.05) (Table 5). Lysosomal activity, phagocytic activity, and phagocytic index are shown in Table 5. The obtained results showed that in ovo feeding with clenbuterol 10 and 15 ppm in embryos led to a regular immune pattern in other treated groups.

### 3.4. Muscle Fatty and Amino Acid Profiles

Table 6 shows that the in ovo feeding of clenbuterol at 10 and 15 ppm resulted in significantly decreased (*p* < 0.05) low saturated fatty acids (myristic, palmitoleic, stearic, and palmitic) contents of the breast muscles compared to the other treated groups. Meanwhile, the current study revealed no significant variations of the in ovo feeding of clenbuterol to modulate the polyunsaturated FA (PUFA) contents in the breast muscles in other treated groups, including α-linolenic acid linoleic acid, docosahexaenoic acid, and eicosapentaenoic acid. As shown in Table 7, the in ovo feeding of clenbuterol at 10 and 15 ppm showed a marked increase in lysine, threonine, leucine, phenylalanine, methionine, valine, and isoleucine muscular contents compared with the control and normal saline groups. Moreover, in ovo feeding of clenbuterol significantly increased the nonessential AA contents in chicken breast muscles, such as serine, alanine, arginine, proline, and aspartic acid, especially at 15 ppm concentration in other treated groups.

**Figure 1:**
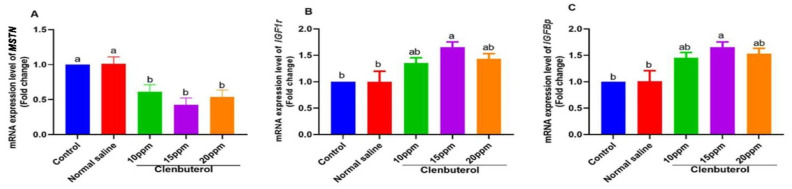
Expression of fold changes of Muscular (**A**) *MSTN*, Myostatin gene, (**B**) *IGF1R*, insulin growth factor1 receptor, (**C**), *IGFBP2*, Insulin-like growth factor-binding protein 2. Data Mean ± SE were analyzed with one-way ANOVA followed by Duncan’s multiple comparison test. Columns with differ superscripts (a, b, c, d) differ significantly (*p* < 0.05).

**Figure 2:**
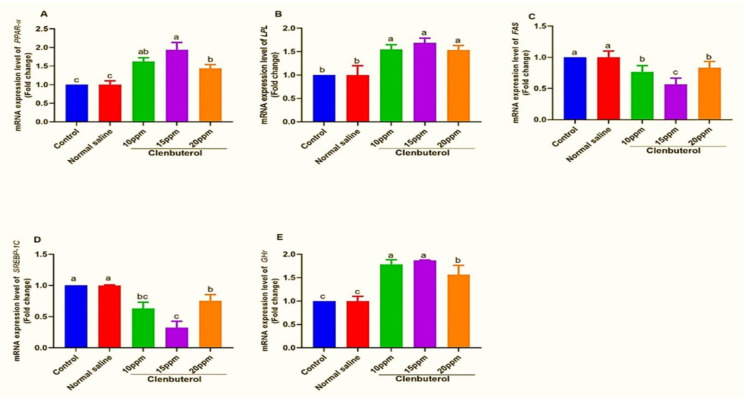
Expression of fold changes of hepatic (**A**) *PPARα*, peroxisome proliferator-activated receptors, (**B**) *LPL*, Lipoprotein lipase, (**C**) *FAS*, Fatty acid synthase, (**D**) *SREBP-1c*, Sterol regulatory element-binding protein 1, (**E**) *GHR*, growth hormone receptor. Data Mean ±SE were analyzed with one-way ANOVA followed by Duncan’s multiple comparison test. Columns with differ superscripts (a, b, c, d) differ significantly (*p* < 0.05).

Figure 1 shows that in ovo feeding of clenbuterol led to marked downregulation and myostatin gene expression in the breast muscle (*p* < 0.01) in the control and normal saline groups. Clenbuterol at 15 ppm showed significant upregulation of *IGF1r*, and clenbuterol significantly upregulated *IGFBP* expression at 15 ppm concentration in other treated groups; in the same context, clenbuterol feeding showed significant upregulation of hepatic *PPARα* in other treated groups (*p* < 0.05, *p* < 0.01, *p* < 0.05) for clenbuterol at 10 ppm, 15 ppm, and 20 ppm, respectively. Clenbuterol feeding showed significant upregulation in *LPL* mRNA expression with a marked decrease in *FAS* mRNA expression. Moreover, there was significant downregulation in *SREBP-1c* expression in clenbuterol groups in other treated groups with marked upregulation in *GHr* expression, as shown in Figure 2.

### 3.5. Histomorphometry of the Duodenum, Spleen and Bursa of Fabricius

**Figure 3:**
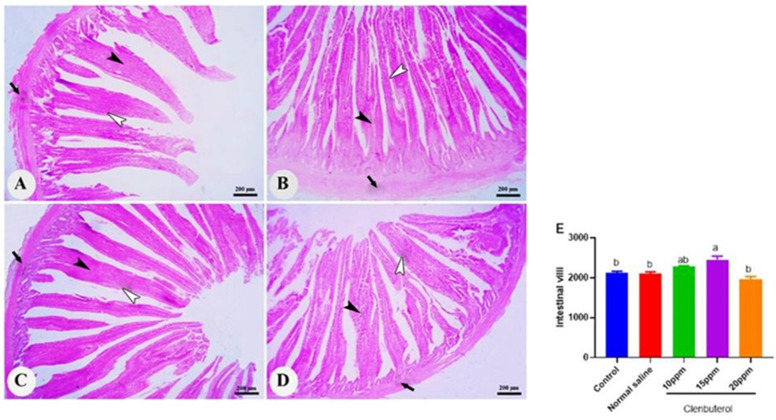
Photomicrograph of duodenum of control (**A**) and clenbuterol treated groups of chickens; (**B**) clenbuterol 10 ppm, (**C**) clenbuterol 15 ppm and (**D**) clenbuterol 20ppm showing intestinal villi composed of simple columnar epithelium (white arrow heads), connective tissue core (black arrow heads) of lamina propria and lamina muscularis (black arrows). Stain H&E, scale bar= 200 µm. (**E**) morphometric analysis of duodenal villi length. Columns with differ superscripts (a, b) differ significantly (*p* < 0.05).

**Figure 4:**
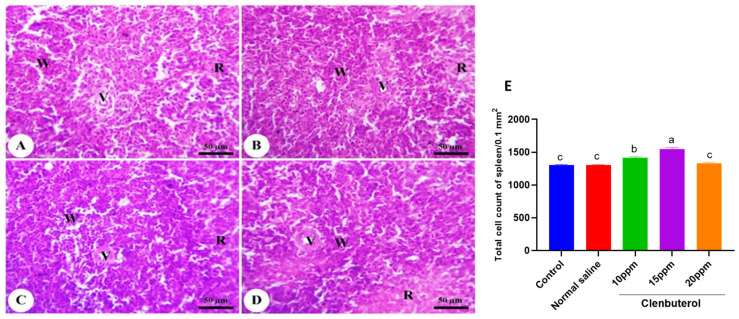
Photomicrograph of spleen of control (**A**) and clenbuterol treated groups of chickens; (**B**) clenbuterol 10 ppm, (**C**) clenbuterol 15 ppm and (**D**) clenbuterol 20 ppm showing white pulp (W) surrounding an artery (V) and loosely arranged blood sinusoids forming the red pulp (R). Stain H&E, scale bar = 50 µm. (**E**) morphometric analysis of total cell count of splenic parenchyma. Columns with differ superscripts (a, b, c, d) differ significantly (*p* < 0.05).

**Figure 5:**
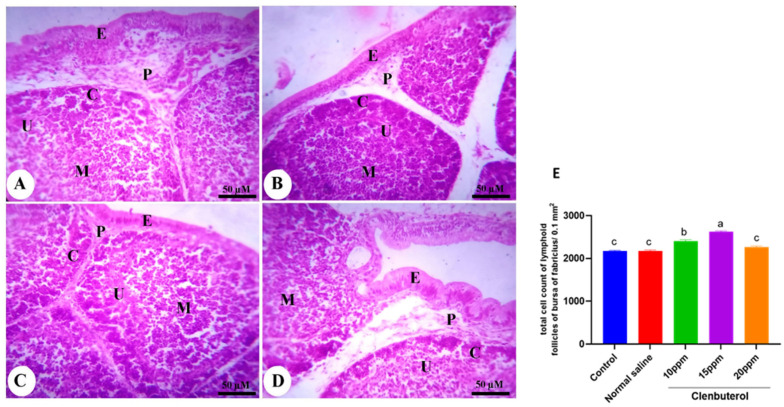
Photomicrograph of the bursa of Fabricius of control (**A**) and clenbuterol treated groups of chickens; (**B**) clenbuterol 10 ppm, (**C**) clenbuterol 15 ppm and (**D**) clenbuterol 20 ppm showing pseudostratified columnar epithelium (**E**), lamina propria (P), cortex (C) and medulla (M) of lymphoid follicle separated by undifferentiated cell layer (U). Stain H&E, scale bar = 50µm. E. morphometric analysis of total cell count of lymphoid follicles of the bursa of Fabricius. Columns with differ superscripts (a, b, c, d) differ significantly (*p* < 0.05).

The duodenum of the control group was formed of mucosa, submucosa, muscularis, and serosa. The mucosa was thrown into the intestinal villi in the intestinal lumen and mucosal glands in the lamina propria. The intestinal villi became more numerous and branched, in addition to a significant increase in villi length (*p* < 0.001) in the clenbuterol 10 ppm group compared with the control and clenbuterol 15 ppm groups (Figure 3). The normal saline in ovo injected group showed no difference from the control group. The spleen of the chicken was formed of white and red pulp. The white pulp was composed of lymphatic follicles and periarterial lymphoid sheath. The red pulp was formed of blood sinusoid and blood cells. The lymphatic nodule contained blood vessels. The fourth group’s spleen revealed a marked increase in the number of small lymphocytes in addition to the size of lymphatic nodules in the clenbuterol 10 ppm and clenbuterol 15 ppm groups compared with the other groups (Figure 4). The histopathological examination of the bursa of Fabricius revealed that the mucosal layer was thrown into several folds lined by pseudostratified columnar epithelium. Each fold contained several lymphoid follicles. Each follicle was surrounded by loose connective tissue from the lamina propria—the follicle composed of a darkly stained cortex and lightly stained medulla due to dispersed lymphocytes. The two layers were separated by undifferentiated cells with acidophilic cytoplasm. The cortex and medulla of the clenbuterol 10 ppm and 15 ppm groups were darker than those of the control and clenbuterol 20 ppm groups due to an increase in the density of lymphoid cells (Figure 5).

## 4. Discussion

Clenbuterol has been shown to increase skeletal muscle mass in mammals [60]. Clenbuterol is a selective 2-adrenoceptor agonist with the ability to cross the blood–brain barrier that works by binding to 2-adrenoceptors and activating the enzyme adenylyl cyclase, which causes an increase in intracellular concentrations of cyclic adenosine monophosphate and, as a result, protein kinase A activation [61]. As a result of its numerous adverse effects on humans, such as cardiomyopathy and acute hepatitis, clenbuterol has been banned in several nation [62].

Our study examined the effect of in ovo feeding of clenbuterol on fertility, hatchability, growth performance, and multiple molecular and physiological parameters concerning the pathway by which clenbuterol exerts its action. Table 3 shows the effects of in ovo feeding on growth and carcass traits, in which the clenbuterol-injected group at 15 ppm showed significantly higher weight gain and final body weight, and improved hatchability and fertility percentage. Additionally, there was a considerably enhanced carcass yield and significantly decreased abdominal weight percentage at 10 and 15 ppm doses of clenbuterol in other treated groups. Our obtained result was in agreement with those of previous studies [63,64]. They proved that b-adrenergic agonists could boost weight gain when added to feed, and the proportion of tissue fat is reduced [65]. These findings are attributed to increased nitrogen accrual and deterioration in the saturated fatty acid concentration [66].

Additionally, Spurlock, et al. [67] reported that clenbuterol administration stimulated anabolic activity. All previously mentioned studies support in ovo feeding of clenbuterol findings concerning weight gain and abdominal fat deposition. Our results are consistent with Hamano [68], who reported significant weight decreases in the abdominal fat in chicken fed 0.25 mg/kg of clenbuterol. In the same line, clenbuterol caused decreased abdominal fat [69]. Our histopathological results support clenbuterol’s overall growth success, which may be due to the increased height of the villus in all small intestine segments.

Moreover, with in ovo feeding, clenbuterol has no significant effect on RBC count, Hb level, or H/L ratio, as shown in Table 4. There are no marked changes in normal liver activity enzyme. Similarly, Mohamed et al. [20] found that clenbuterol at five and ten ppm had no significant effect on liver function and white blood cells, reflecting the nonstressful condition of in ovo clenbuterol administration.

In contrast, a significant increase in total protein and albumin levels was observed in the ovo clenbuterol treated group at 10 and 15 ppm. Takahashi et al. [64] found that clenbuterol enhanced the carcass protein, in which the beta-agonist eased protein breakdown and increased the metabolic protein rate [70]. Moreover, Mohamed et al. [20] reported that clenbuterol significantly increased the total protein concentration because it increased protein synthesis and decreased degradation [22].

Lipid markers decreased with in ovo clenbuterol feeding relative to saline feeding and control, as shown in Table 4. Our finding was in harmony with [20] who reported that lipid profile was reduced with clenbuterol administration in fish ascribed to the role of clenbuterol in the impaired synthesis of cholesterol in the liver and body fat adipocytes that affect its release to muscle tissue. Ijiri et al. [22] found that cholesterol decreased in chicks injected with clenbuterol. In ovo feeding of clenbuterol showed no effect on the overall antioxidant activity. Our result was supported by [71], who reported that clenbuterol administration had no significant effect on the antioxidant activity in the control group with an ischemia-induced injury in an isolated rat heart. In ovo feeding of clenbuterol showed no significant difference in phagocytic activity and lysosomal activity in different treated groups. As shown in Table 6, in ovo feeding of clenbuterol at 10 and 15 ppm significantly decreased (*p* < 0.05, *p* < 0.01) lower saturated fatty acid (myristic, stearic, and palmitic) content of the breast muscles compared to other treated groups.

Meanwhile, the current study revealed no significant variations of in ovo feeding of clenbuterol to modulate the PUFA contents in the breast muscles concerning other treated groups: α-linolenic acid, linoleic acid, and docosahexaenoic acid. These data were consistent with [72], in which they reported that beta-adrenergic agonist therapy reduced the unsaturated fatty acids and increased the saturated fatty acids in *M. longissimus Dorsi* steers treated with beta-adrenergic agonist and showed that stearic acid might be negatively cholesterolemic, which decreased the cholesterol level. No previous studies have examined the effect of in ovo clenbuterol feeding on the amino acid contour of chicken muscle. In this context, the current study suggested that, as shown in Table 7, in ovo feeding of clenbuterol at 10 and 15 ppm showed a marked increase in lysine, threonine, leucine, phenylalanine, methionine, valine, and isoleucine muscular contents compared with the control and normal saline groups.

Moreover, in ovo feeding of clenbuterol significantly increased the nonessential amino acid contents in chicken breast muscles, such as serine, alanine, arginine, proline, and aspartic acid at 15 ppm in other treated groups. These data may be accredited to improving the protein content of muscles compared to the control group. Kheiri and Alibeyghi [73] revealed that the carcass yield and growth performance could be upgraded with the increase in lysine and threonine levels, which supports our growth markers.

Additionally, it is crucial to investigate the transcriptomic pathway of clenbuterol concerning muscle growth. It was found that in ovo feeding of clenbuterol led to marked downregulation and myostatin gene expression (*p* < 0.01) in the control and normal saline groups. Similarly, Ijiri et al. [22] found that clenbuterol feeding resulted in decreased muscle myostatin expression. Myostatin is an essential regulator for skeletal muscle growth and contributes to clenbuterol-induced muscle growth and mass [74]. Lalani et al. [75] showed that *IGF-1* had a positive regulatory impact on muscle growth. These reports supported our finding in which clenbuterol at 15 ppm had significant upregulation of *IGF1r*. These findings were consistent with [74,76]. They found that clenbuterol leads to *IGF-1* upregulation. Moreover, Abo et al. [2] showed that an insulin-like growth factor is well established as a fundamental part of embryonic muscle development and proliferation. However, clenbuterol at concentrations of 10 and 20 ppm did not affect the *IGF-1* receptor level. These data were in harmony with [22], who reported that clenbuterol feeding on a one-day-old chick did not change the *IGF-1* expression. These data were also supported by *IGFBP* expression in which clenbuterol significantly upregulates *IGFBP* expression at 15 ppm concentration in other treated groups. These data were in line with [77], who reported that muscle growth stimulated by clenbuterol is coupled with a local increase in muscle *IGFBP* content.

*GHR* is a growth hormone transmembrane receptor, an important hormone for normal growth [78]. Clenbuterol significantly upregulates hepatic *GHr* expression; this result was consistent with those of [79], who reported the importance of growth hormone in the postnatal growth of skeletal muscles, and *IGF-1* and **GHR** both have good growth regulators, which leads to anabolic effects on proteins and carbohydrate metabolism, and mediates growth hormone activity [80]. In the same context, clenbuterol feeding led to significant upregulation of *PPARα* in other treated groups (*p* < 0.05), (*p* < 0.01), (*p* < 0.05) for clenbuterol at 10 ppm, 15 ppm, and 20 ppm, respectively. *PPARα* is highly expressed in the liver and plays a key role in lipid metabolism-boosting fatty acid oxidation [81]. This supports our finding that in ovo clenbuterol feeding possibly lessens fat deposition by the oxidation of long-chain fatty acids in the mitochondria and fatty acid oxidation in the liver [82]. Clenbuterol feeding showed significant upregulation in *LPL* mRNA expression with markedly decreased *FAS* mRNA expression, and this was consistent with [20], who reported that clenbuterol upregulated expression of the liver *LPL* gene. Clenbuterol modulates mRNA expression levels through attenuation of lipogenic activity (downregulated levels of the *FAS* in the liver) and fatty acid oxidation (increased levels of *LPL* in the liver gene expression), which facilitate lipid catabolism in agreement with our results. Kim et al. [82] reported that clenbuterol increased the rate of lipolysis and decreased the lipogenesis rate for adipose tissues.

Moreover, there was a significant downregulation in *SREBP-1c* expression in clenbuterol groups than in other treated groups. Sterol regulatory element-binding proteins- 1 and -2 (*SREBP-1 and -2*) are key transcript components implicated in cholesterol and fatty acid biosynthesis. Our data were in harmony with [9]. They reported that clenbuterol reduced the *SREBP-1c* expression, which supports our earlier result.

## 5. Conclusions

In ovo feeding of clenbuterol improved hatchability, fertility, and growth efficiency, promoted lipolysis, modulated lipid markers, and decreased abdominal fat. Moreover, clenbuterol enhanced poultry body gain via upregulation of insulin growth factor 1 receptor and insulin-like growth factor-binding protein 2 expression, downregulated myostatin gene, and increased protein synthesis. In addition, clenbuterol increased the intestinal villi without significant alterations in the histopathology of the bursa of Fabricius and spleen. The in ovo clenbuterol feeding led to higher oxidation of fatty acids and increased growth weight.

## Figures and Tables

**Table 1:** Ingredients and calculated chemical composition of the basal diet (g/kg).

Ingredients	Experimental Diets	
	Starter (0–4 weeks)	Grower (5–12 weeks)
Yellow corn	55.3	60.33
Soybean meal, 48%	38.47	33.1
Soybean oil	2.05	2.40
Di-calcium phosphate	1.73	1.75
Limestone	1.25	1.05
Mineral and Vitamin premix ^1^	0.3	0.3
Salt	0.22	0.22
Sodium bicarbonate	0.32	0.25
DL-methionine	0.18	0.20
Lysine	0.19	0.40
Nutrients Composition		
Metabolizable energy (Kcal/kg)	3000	3050
Crude protein%	23.04	21.05
Crude fat%	4.51	4.97
Lysine%	1.49	1.47
Methionine%	0.56	0.55
Calcium%	0.96	0.88
Available phosphorous	0.44	0.42
Sodium	0.2	0.18

^1^ Mineral and Vitamin premix produced by Multi Vita provides vitamin A 12,000 IU, vitamin D3 2500 IU, vitamin E 20 mg, vitamin K3 2 mg, vitamin B1 2 mg, vitamin B2 5 mg, vitamin B6 2 mg, vitamin B12 0.05 ug, niacin 30 mg, biotin 0.05 ug, folic acid 1 mg, pantothenic acid 10 mg, manganese 60 mg, zinc 50 mg, iron 40 mg, copper 10 mg, iodine 0.6 mg, selenium 0.3 mg per 1 kg diet. DL-Methionine (Produced by Evonic Co. and contains 99% methionine). Lysine = lysine hydrochloride (Produced by Evonic Co. contains 70% Lysine).

**Table 2:** Primers for gene expression by RT-PCR GAPDH.

Gene	Forward	Reverse	Accession Number	Amplicon (pb)	References
*GAPDH*	ACATGGCATCCAAGGAGTGAG	GGGGAGACAGAAGGGAACAGA	NM_204305	158	[51]
*IGF1R*	TTCAGGAACCAAAGGGCGA	TGTAATCTGGAGGGCGATACC	NM_205032	167	[52]
*PPARα*	TGTGGAGATCGTCCTGGTCT	CGTCAGGATGGTTGGTTTGC	NM_001001464	103	[51]
*SREBP-1c*	TCACCGCTTCTTCGTGGAC	CTGAAGGTACTCCAACGCATC	AY029224	220	[51]
*FAS*	CAATGGACTTCATGCCTCGGT	GCTGGGTACTGGAAGACAAACA	NM_205155.2	119	[51]
*LPL*	GTGACCAAGGTAGACCAGCC	GAAGAGACTTCAGGCAGCGT	NM_205282.1	62	[53]
*MSTN*	TTACCCAAAGCTCCTCCACTG	AGGATCTGCACAAACACCGT	NM_001001461	120	[54]
*GHR*	CATGGCCACCTTTTGCAGAC	ACCTTGGATTTCTGCCCTGG	NM_001001293	121	[55]
*IGFBP2*	CACAACCACGAGGACTCAAA	CATTCACCGACATCTTGCAC	NM_205359.1	299	[56]

Glyceraldehyde-3-phosphate dehydrogenase. *IGF1R*, insulin growth factor1 receptor, *PPARα*, peroxisome proliferator-activated receptors, *SREBP-1c*, Sterol regulatory element-binding protein 1, *FAS*, Fatty acid synthase, *LPL*, Lipoprotein lipase, *MSTN*, Myostatin gene, *GHR*, growth hormone receptor, *IGFBP2*, Insulin-like growth factor-binding protein 2.

**Table 3:** Effect of in ovo feeding of clenbuterol on growth performance pattern of chicken.

Parameters	Control	Normal Saline	Clenbuterol			*p*-Value	Polynomial Contrasts	
			10 ppm	15 ppm	20 ppm		Linear	Quadratic
Hatchability (%)	84 ± 1.3 ^b^	82.6 ± 2.2 ^b^	89.9 ± 3.2 ^a^	90.2 ± 1.5 ^a^	90.3 ± 3.4 ^a^	0.046	0.012	0.001
IBW (g/bird)	31.1 ± 0.4	31.1 ± 1.4	30.3 ± 0.4	30.1 ± 0.8	30.2 ± 0.7	0.110	0.114	0.223
FBW (g/bird)	2680.1 ± 29.2 ^b^	2679.5 ± 33.5 ^b^	2815.5 ± 17.2 ^a^	2938.2 ± 25.7 ^a^	2655.4 ± 25.8 ^b^	0.021	0.001	0.024
WG (g/bird)	2649.2 ± 25.5 ^b^	2648.4 ± 3.4 ^b^	2785.2 ± 3.5 ^ab^	2908.0 ± 7.4 ^a^	2625.2 ± 3.7 ^b^	0.001	0.01	0.0254
FCR (g feed/gain)	1.6 ± 3.2	1.59 ± 4.8	1.52 ± 4.3	1.47 ± 3.4	1.65 ± 7.2	0.165	0.021	0.214
Carcass (%)	72.5 ± 1.2 ^b^	71.5 ± 0.4 ^b^	73.15 ± 3.1 ^b^	75.54 ± 2.1 ^a^	72.15 ± 1.4 ^b^	0.002	0.015	0.014
Abdominal Fat (% of eviscerated weight)	2.7 ± 0.1 ^a^	2.75 ± 0.1 ^a^	2.014 ± 0.2 ^b^	1.86 ± 0.1 ^c^	2.11 ± 0.1 ^b^	0.002	0.014	0.018

Means ± SEM displaying different superscript letters are significantly (*p* < 0.05) different from the other values within the same raw (between groups). IW = Initial body weight. FW = Final body weight, FCR = feed conversion ratio.

**Table 4:** Effect of in ovo feeding of clenbuterol on hematological and biochemical markers pattern of chicken.

Parameters	Control	Normal Saline	Clenbuterol			*p*-Value	Polynomial Contrasts	
			10 ppm	15 ppm	20 ppm		Linear	Quadratic
RBC (×10^6^/μL)	4.01 ± 0.32	3.91 ± 0.32	3.75 ± 0.14	3.71 ± 0.21	3.47 ± 0.14	0.125	0.214	0.31
Hemoglobin (g/dL)	12.05 ± 0.21	11.88 ± 0.3	11.52 ± 0.32	12.14 ± 0.5	11.50 ± 0.21	0.121	0.125	0.25
H/L ratio%	0.56 ± 0.01	0.57 ± 0.04	0.59 ± 0.14	0.60 ± 0.15	0.62 ± 0.32	0.11	0.31	0.45
Cholesterol (mg/dL)	110.54 ± 5.4 ^a^	112.5 ± 1.3 ^a^	82.14 ± 1.4 ^c^	79.15 ± 2.3 ^bc^	85.05 ± 3.2 ^b^	0.241	0.01	0.035
TG (mg/dL)	103.15 ± 6.2 ^a^	106.4 ± 4.4 ^a^	92.45 ± 3.4 ^c^	85.45 ± 2.4 ^d^	101 ± 2.1 ^b^	0.014	0.021	0.01
Total lipids (mg/dL)	198.9 ± 4.1 ^a^	200.1 ± 2.2 ^a^	156.36 ± 3.4 ^c^	150.05 ± 3.1 ^c^	161.56 ± 4.1 ^b^	0.018	0.015	0.021
HDL-C (mg/dL)	55.1 ± 2.1	54.2 ± 1.4	57.6 ± 2.1	59.45 ± 2.5	56.48 ± 1.1	0.144	0.144	0.21
LDL-C (mg/dL)	79.45 ± 3.4 ^a^	79.15 ± 2.1 ^a^	75.58 ± 2.1 ^c^	74.45 ± 2.1 ^c^	78.15 ± 1.4 ^b^	0.001	0.014	0.014
Albumin (g/dL)	1.92 ± 0.2 ^b^	1.99 ± 0.14 ^b^	2.12 ± 0.24 ^a^	2.14 ± 0.3 ^a^	1.95 ± 0.1 ^b^	0.001	0.001	0.01
Globulin (g/dL)	2.12 ± 0.14	2.10 ± 0.1	2.15 ± 0.5	2.20 ± 0.3	2.10 ± 0.4	0.125	0.14	0.12
Total protein (g/dL)	4.04 ± 0.15 ^b^	4.09 ± 0.14 ^b^	4.27 ± 0.3 ^a^	4.34 ± 0.4 ^a^	4.05 ± 0.5 ^b^	0.011	0.02	0.001
AST (U/L)	112.10 ± 4.1	110.15 ± 2.0	111.05 ± 3.0	115.45 ± 3.1	116.48 ± 4.1	0.125	0.45	0.32
ALT (U/L)	12.15 ± 0.4	12.23 ± 1.2	10.95 ± 0.8	11.02 ± 0.9	11.47 ± 0.4	0.154	0.158	0.28
TAC (U/L)	1.81 ± 0.2	1.83 ± 0.2	1.82 ± 0.1	1.83 ± 0.3	1.80 ± 0.4	0.121	0.147	0.14

Means ± SEM displaying different superscript letters are significantly (*p* < 0.05) different from the other values within the same raw (between groups). H/L ratio, Heterophil/lymphocyte ratio. TG, triglyceride. HDL, High-density lipoprotein. LDL, low density lipoprotein. AST, Aspartate transaminase. ALT, Alanine transaminase. TAC, Total antioxidant capacity.

**Table 5:** Effect of in ovo feeding of clenbuterol on the antioxidants and immunological markers pattern.

Parameters	Control	Normal Saline	Clenbuterol			*p*-Value	Polynomial Contrasts	
			10 ppm	15 ppm	20 ppm		Linear	Quadratic
GSH-PX(U/g)	25.14 ± 1.2	25.22 ± 1.2	26.45 ± 2.1	26.48 ± 1.2	24.91 ± 1.8	0.114	0.112	0.51
MDA (nmol/g)	10.155 ± 1.1	10.78 ± 1.1	10.19 ± 1.1	10.48 ± 1.4	10.54 ± 0.5	0.125	0.102	0.32
SOD (U/mg protein)	45.45 ± 2.0	46.112 ± 2.1	45.47 ± 3.2	46.2 ± 2.1	44.78 ± 2.4	0.214	0.25	0.15
Phagocytic activity (%)	52.15 ± 2.4	51.14 ± 2.0	54.78 ± 1.4	55.78 ± 0.3	52.45 ± 1.4	0.101	0.45	0.21
Phagocytic index	3.42 ± 10.4	3.32 ± 0.1	3.30 ± 1.2	3.56 ± 0.3	3.22 ± 0.4	0.15	0.25	0.45
Lysozyme-activity (μ/mL)	0.89 ± 0.04	0.92 ± 0.1	0.93 ± 0.4	0.91 ± 0.4	0.90 ± 0.02	0.145	0.27	0.32

Data expressed as Mean ± SEM. GSH-PX, Glutathione peroxidase. MDA, Malondialdehyde. SOD, Superoxide dismutase.

**Table 6:** Effect of in ovo feeding of clenbuterol on the breast muscle fatty acid profile (g/100 g fat) of chicken.

Fatty Acids	Control	Normal Saline	Clenbuterol			*p*-Value	Polynomial Contrasts	
			10 ppm	15 ppm	20 ppm		Linear	Quadratic
SFA								
C16:0	29.24 ± 2.1 ^a^	29.12 ± 1.2 ^a^	25.3 ± 1.1 ^b^	20.14 ± 1.1 ^c^	25.4 ± 1.3 ^b^	0.214	0.001	0.21
C16:1	3.75 ± 0.5 ^a^	3.74 ± 0.3 ^a^	3.53 ± 0.4 ^b^	3.62 ± 0.4 ^b^	3.60 ± 0.02 ^b^	0.001	0.01	0.023
C18:0	9.45 ± 0.8 ^a^	9.42 ± 0.7 ^a^	7.48 ± 0.7 ^bc^	6.34 ± 0.3 ^c^	8.65 ± 0.5 ^b^	0.001	0.012	0.04
C14:0	1.18 ± 0.1 ^a^	1.115 ± 0.3 ^a^	0.93 ± 0.02 ^bc^	0.75 ± 0.02 ^c^	1.02 ± 0.01 ^b^	0.001	0.01	0.01
MUFA								
C18:1	18.24 ± 1.1	18.12 ± 0.7	18.11 ± 1.0	18.04 ± 1.1	18.36 ± 0.9	0.145	0.63	0.81
PUFA								
C18:2	23.14 ± 1.2	23.22 ± 2.1	23.33 ±1.2	23.40 ± 1.1	23.45 ± 1.2	0.121	0.365	0.75
C18:3n3	1.62 ± 0.3	1.60 ± 0.4	1.68 ± 0. 1	1.65 ± 0.4	1.64 ± 0.1	0.132	0.225	0.89
C18:2n6	0.92 ± 0.04	0.90 ± 0.07	0.88 ± 0.1	0.86 ± 0.1	0.89 ± 0.1	0.116	0.23	0.52
C22:6n3	0.84 ± 0.01	0.88 ± 0.08	0.82 ± 0.2	0.79 ± 0.1	0.80 ± 0.1	0.132	0.14	0.95

SFA, Saturated fatty acids (Palmitic acid, C16:0. Palmitoleic acid, C16:1. Stearic acid, C18:0. Myristic acid, C14:0). MUFA, Monounsaturated (Oleic acid, C18:1). PUFA, Poly unsaturated (Linoleic acid, C18:2. α-linolenic acid, C18:3n3. γ-linolenic acid, C18:2n6. Docosahexaenoic acid C22:6n3). Means ± SEM displaying different superscript letters are significantly (*p* < 0.05) different from the other values within the same raw (between groups).

**Table 7:** Effect of in ovo feeding of clenbuterol on the breast muscle amino acid profile (g/100 g) of chicken breast muscle.

Amino Acids	Control	Normal Saline	Clenbuterol		
			10 ppm	15 ppm	20 ppm
Leucine	6.76 ± 0.5 ^b^	6.77 ± 0.4 ^b^	6.85 ± 0.8 ^b^	6.89 ± 0.7 ^a^	6.79 ± 0.2 ^b^
Isoleucine	3.28 ± 0.1 ^b^	3.20 ± 0.1 ^b^	3.43 ± 0.1 ^a^	3.52 ± 0.1 ^a^	3.30 ± 0.1 ^b^
Phenylalanine	2.12 ± 0.3 ^c^	2.10 ± 0.1 ^c^	2.55 ± 0.1 ^a^	2.69 ± 0.1 ^a^	2.20 ± 0.1 ^b^
Lysine	7.30 ± 0.8 ^b^	7.29 ± 0.9 ^b^	7.45 ± 0.3 ^a^	7.50 ± 0.5 ^a^	7.42 ± 0.8 ^a^
Methionine	2.14 ± 0.1 ^b^	2.22 ± 0.1 ^b^	2.30 ± 0.2 ^a^	2.39 ± 0.3 ^a^	2.20 ± 0.1 ^b^
Valine	4.02 ± 0.2 ^c^	4.1 ± 0.1 ^c^	4.22 ± 0.1 ^a^	4. 33 ± 0.4 ^a^	4.11 ± 0.1 ^b^
Threonine	3.75 ± 0.1 ^b^	3.70 ± 0.2 ^b^	3.90 ± 0.2 ^a^	3.95 ± 0.1 ^a^	3.72 ± 0.1 ^b^
Serine	3.51 ± 0.1 ^c^	3.55 ± 0.2 ^c^	3.69 ± 0.2 ^b^	3.77 ± 0.1 ^a^	3.52 ± 0.1 ^c^
Glycine	3.42 ± 0.4	3.44 ± 0.2	3.44 ± 0.3	3.40 ± 0.1	3.33 ± 0.2
Glutamic acid	11.55 ± 0.4	11.45 ± 0.7	11.57 ± 0.8	11.53 ± 0.6	11.49 ± 0.4
Aspartic acid	8.3 ± 0.3 ^c^	7.98 ± 0.2 ^c^	8.15 ± 0.4 ^b^	8.20 ± 0.4 ^a^	8.10 ± 0.8 ^bc^
Alanine	4.92 ± 0.2 ^b^	4.88 ± 0.4 ^b^	4.95 ± 0.1 ^a^	5.05 ± 0.4 ^a^	4.89 ± 001 ^b^
Tyrosine	2.81 ± 0.1	2.80 ± 0.2	2.82 ± 0.3	2.89 ± 0.2	2.83 ± 0.5
Cysteine	1.69 ± 0.1	1.73 ± 0.1	1.72 ± 0.3	1.75 ± 0.3	1.68 ± 0.3
Histidine	3.15 ± 0.3	3.22 ± 0.4	3.12 ± 0.2	3.22 ± 0.2	3.19 ± 0.3
Arginine	5.14 ± 0.2 ^c^	5.12 ± 0.4 ^c^	5.22 ± 0.2 ^b^	5.35 ± 0.1 ^a^	5.12 ± 0.2 ^c^
Proline	2.19 ± 0.1 ^b^	2.24 ± 0.1 ^b^	2.21 ± 0.1 ^b^	2.41 ± 0.2 ^a^	2.18 ± 0.1 ^b^

Means ± SEM displaying different superscript letters are significantly (*p* < 0.05) different from the other values within the same raw (between groups).

## Data Availability

Data are available upon request.
